# Topical Olive Oil Is Not Inferior to Hyperoxygenated Fatty Aids to Prevent Pressure Ulcers in High-Risk Immobilised Patients in Home Care. Results of a Multicentre Randomised Triple-Blind Controlled Non-Inferiority Trial

**DOI:** 10.1371/journal.pone.0122238

**Published:** 2015-04-17

**Authors:** Inmaculada Lupiañez-Perez, Shakira Kaknani Uttumchandani, Juan Carlos Morilla-Herrera, Francisco Javier Martin-Santos, Magdalena Cuevas Fernandez-Gallego, Francisco Javier Navarro-Moya, Yolanda Lupiañez-Perez, Eugenio Contreras-Fernandez, Jose Miguel Morales-Asencio

**Affiliations:** 1 Malaga-Guadalhorce Primary Healthcare District, Andalusian Health Service, Malaga, Spain; 2 Faculty of Health Sciences, University of Malaga, Malaga, Spain; 3 Virgen de la Victoria University Hospital and Carlos Haya Regional Hospital, Andalusian Health Service, Malaga, Spain; 4 Costa del Sol Primary Healthcare District, Andalusian Health Service, Malaga, Spain; Weill Cornell Medical College Qatar, QATAR

## Abstract

Pressure ulcers represent a major current health problem and produce an important economic impact on the healthcare system. Most of studies to prevent pressure ulcers have been carried out in hospital contexts, with respect to the use of hyperoxygenated fatty acids and to date, no studies have specifically examined the use of olive oil-based substances.

**Methods and Design:**

Main objective: To assess the effectiveness of the use of olive oil, comparing it with hyperoxygenated fatty acids, for immobilised home-care patients at risk of suffering pressure ulcers. Design: Non-inferiority, triple-blind, parallel, multicentre, randomised clinical trial. Scope: Population attending Primary Healthcare Centres in Andalusia (Spain). Sample: 831 immobilised patients at risk of suffering pressure ulcers.

**Results:**

The follow-up period was 16 weeks. Groups were similar after randomization. In the per protocol analysis, none of the body areas evaluated presented risk differences for pressure ulcers incidence that exceeded the 10% delta value established. Sacrum: Olive Oil 8 (2.55%) vs HOFA 8 (3.08%), ARR 0.53 (-2.2 to 3.26) Right heel: Olive Oil 4 (1.27%) vs HOFA 5 (1.92)%, ARR0.65 (-1.43 to 2.73). Left heel: Olive Oil 3 (0.96%) vs HOFA 3 (1.15%), ARR0.2 (-1.49 to 1.88). Right trochanter: Olive Oil 0 (0%) vs HOFA 4 (1.54%), ARR1.54 (0.04 to 3.03). Left trochanter: Olive Oil 1 (0.32%) vs HOFA 1 (0.38%), ARR0.07 (-0.91 to 1.04). In the intention to treat analysis the lower limit of the established confidence interval was never exceeded.

**Discussion:**

The results obtained confirmed that the use of topical extra-virgin olive oil to prevent PU in the home environment, for immobilised patients at high risk, is not inferior to the use of HOFA. Further studies are needed to investigate the mechanism by which olive oil achieves this outcome.

**Trial Registration:**

Clinicaltrials.gov NCT01595347

## Introduction

Pressure ulcers (PU) provoke healthcare, economic and social problems. They produce a significant deterioration in patients’ quality of life [1], with a considerable physical, social, psychological and economic impact and deterioration in overall health, with the consequent reduction in life expectancy. Moreover, informal caregivers of these patients are stressed by a challenging workload, which in turn provokes numerous pathologies [2]. PU represent the alteration of a basic need, that of maintaining the integrity of the skin. They can appear anywhere on the body, but are most common on bony prominences (the sacrum, hips and heels), and are suffered particularly by elderly patients who are immobilised with severe acute disease and a neurological deficit. A PU is a skin lesion produced secondarily to a process of ischemia. It can lead to necrosis in areas of the epidermis, dermis, subcutaneous tissue and muscle where it is present, and may even affect the joints and bones. It tends to occur when the soft tissue is compressed between two planes, one of which is the bony prominence of the patient and the other, an external surface [3].The development of PU is often accompanied by vascular occlusion, due to the external pressure and by endothelial damage affecting microcirculation and the arterioles, primarily due to the effect of tangential forces, shear and friction.

The incidence and prevalence of PU are among the most representative indicators of the quality of nursing care. The prevalence data for hospitals in Spain (8.24%) [4] are very similar to those for neighbouring countries such as Italy (8.3%), France (8.9%), Germany (10.2%) and Portugal (12.5%) [5,6], and also to those of more distant countries, such as Jordan (12%) [7].The highest prevalence values reported correspond to Ireland (18.5%), Wales (26.7%) [8], Belgium (21.1%), United Kingdom (21.9%), Denmark (22.7%) and Sweden (23.0%) [9]. With respect to the occurrence of PU in homes for the elderly, perhaps the most important study carried out is that by Park-Lee [10], who analysed the situation of nursing homes in the USA and recorded a prevalence of 11%, similar to that found in Spain. In primary healthcare, in patients receiving home care, there are no data to compare the prevalence in Spain with that in other countries because the characteristics of health care systems vary greatly from country to country. However, we do have data from the second National Survey of Prevalence of Pressure Ulcers in Spain in 2005 [11], in which the crude prevalence of PU was 3.73% and the average prevalence recorded was 9.11% ± 10.9% for patients aged over 14 years and included in the Home Care programme; in the third such National Study, in 2009 [4], the crude prevalence was 5.89%. This increase reflects the aging of the population in Spain during this period.

PU currently represents a major health problem. They produce a severe economic impact and significantly increase pharmaceutical expenditure. The cost of treating PU amounts to 5% of total annual health expenditure [12], and they impose a heavy burden on healthcare personnel.

The major risk factors involved in the occurrence of PU are the mechanical forces—pressure, friction and shear—and the intrinsic and extrinsic factors that tend to decrease tissue tolerance to these forces—immobility, incontinence, malnutrition and reduced level of consciousness. Coleman [13] recently developed a new conceptual framework for studying PU, incorporating physiological and biomechanical components and their impact on internal tensions, together with individual susceptibility and tolerance to these changes.

PU are an indirect indicator of the quality of care. Thus, a low rate of appearance of PU is synonymous with high-quality nursing care with effective preventive measures. The assessment of risk factors, the use of support surfaces, repositioning the patient, maintaining optimal nutritional status, hydration and skin care are all valuable strategies to prevent PU [14–19].

For skin care, the pharmaceutical industry has launched products such as oils derived from hyperoxygenated fatty acids (HOFA). Various studies [20–25] have confirmed the effectiveness of HOFA in maintaining skin integrity and preventing the formation of PU or delaying their onset. Gallart [25] reported an incidence rate of 19% in the target group (preventive measures + HOFA) compared to 35% in the placebo group (preventive measures alone) (p<0.007), RAR = 16 (95%CI: 3.9 to 28.1), RRR = 45.7 (95%CI: 11.8 to 66.6) and NNT = 7 (95%CI: 4–26). In another study, TorraiBou[21]reported an incidence of PU of 7.32% in the target group (Mepentol HOFA) versus 17.37% in the placebo group (oily placebo) (p<0.001), with RAR = 10 (95%CI: 3.1 to 17.0), RRR = 57.9 (95%CI: 20.3 to 77.7) and NNT = 10 (95%CI: 6–33).

One disadvantage of this treatment is its high cost, especially when prolonged treatment is needed to prevent PU. Most studies of effectiveness in the prevention of PU are performed in hospitals or closed institutions and primarily consider the use of compounds based on HOFA.

An alternative could be olive oil. This product is rich in oleic acid, has an important concentration of natural antioxidants (such as hydroxytyrosol and tyrosol), and a high resistance to oxidative processes. Olive oil has between 330 to 500 mg of polyphenolsperkilogram of fat, and under 20mEq of peroxide perkilogram of fat. It is compatible with human tissues what converts it in an ideal product for topic use[26,27]. Consequently, we hypothesised that this alternative treatment could achieve a similar effect, with respect to preventing PU, to products based on HOFA, but at a much lower cost. To resolve this question, a non-inferiority study design is considered the most appropriate. Furthermore, there is no history of experimentation with this product for the prevention of PU in home care patients.

In summary, the aim of this study is to determine whether the use of olive oil achieves results that are not inferior to those obtained by HOFA in preventing PU in immobilised home care patients.

## Methods

The protocol for this trial and supporting CONSORT checklist are available as S1 CONSORT Checklist and S1 Protocol.

### Trial Design

The study design adopted was that of a non-inferiority, triple-blind, parallel, multicentre, randomised clinical trial. The protocol of the study is described in Trials [28], and the study CONSORT checklist is available as S1 CONSORT Checklist and S1 Protocol.

Two procedures were performed: usual care, with the application of HOFA to the control group, and usual care with the application of an olive-oil composition to the target group. The main outcome was the appearance of Stage 2 or higher PU. The accuracy of the trial with regard to the control product (HOFA) is widely supported by historical favourable results of HOVA vs a placebo [21], as it is recommended by the International Conference on Harmonisation. The HOFA efficacy conditions remained intact during the trial.

### Recruitment and selection

The target population were patients included in the immobilised patients programme receiving the home nursing service provided by health centres in Andalusia (Spain).The characteristics of the patients included in the study population were similar to those of the subjects examined in HOFA efficacy trials [21, 25].

The following inclusion criteria were applied: patients aged over 18 years, aided by a family member or paid caregiver for treatment application; risk of impaired skin integrity according to the Braden Scale ≤16, identified by a nurse, and nutritional status of ≤10 according to the Mini Nutritional Assessment (MNA).

Patients were excluded if they refused to take part in the trial, if their permanent address was outside the catchment area of the corresponding health centre, or if they planned to be elsewhere during the follow-up period, if they required hospitalisation during the sampling period, if they were terminally ill or if they already had PU.

All the patients included in the trial received written and verbal information about the aims and characteristics of the study, before they gave written informed consent. When the patients had a cognitive impairment, their legal representative signed the consent form.

### Ethics Statement

Committee accredited by the Decree of 10 June 2011 for Quality General Direction, Research and Knowledge management board Andalusia. Meets 439/2010 decree of 14 December, by which the organs of Healthcare Ethics and Biomedical Research in Andalusia and Decree 223/2004 and BPC standard (CPMP / ICH / 135/95) regulates.

This study was approved by the ethics committee of Malaga Provincial.

### Informed Consent

We obtained informed consent from all participants involved in our study. The consent was written.

#### Sample selection

The directors and care service coordinators of each health centre were informed about the trial, and were asked to confirm their availability and willingness to take part. The community nurses at each health centre were responsible for the practical implementation of the research study and for subsequent follow up, in their respective health centres.

Compliance with the inclusion requirements was checked by examining the electronic clinical records of patients included in the home care programme for immobilised patients. After patients and caregivers had given their informed consent to take part in the study, patients were randomly allocated to a 1:1 control/target group scheme by a computer system blinded to the professionals and the research team. When a patient met the inclusion criteria, his/her nurse was informed of the group to which they allocated by a telephone call from a centralised randomisation unit.

### Outcome measures

The main outcome was the incidence of Stage 2 PU during the 16-week follow-up period. The presence/absence of PU was confirmed by inspection of the areas where the product had been applied (sacrum, hips and heels).

Variables to reflect the characteristics of patients and caregivers were included to assess socio-demographic data, such as the time registered in the care service for immobilised patients, information about previous cases of PU, comorbidities, nutritional and cognitive status, incontinence, ulcer location and availability of technical support devices (mattress or cushions to avoid bedsores, articulated beds, etc.). With respect to the caregivers, age, gender and whether he/she was a family member or paid assistant were determined.

A baseline assessment was performed on all patients at the beginning of the trial, and repeated each week, up to the conclusion of the follow-up period, or until the incidence of any PU.

#### Procedure

All patients, both in the control group and in the target group, were given preventive instructions in accordance with the guidelines to clinical practice on the deterioration of skin integrity, published by the District of Primary Health Care of Malaga. The caregivers in both groups were fully trained to apply the procedure. Together with these preventive measures, the patients in the control group received two applications per day of the HOFA-based product, in the sacral area and on the hips and heels. The HOFA product used presented the CE mark, Class IIb, a higher classification than that of competing products. It was applied topically and included Equisetum Arvense, Hypericum Perforatum and perfume. The olive oil procedure consisted in applying a magistral formula, in liquid spray form, containing 97% extra-virgin olive oil and 3% Hypericum Perforatum and Peppermint. The olives used for this product is the “Picual”. This product had the same appearance as the HOFA. The target group received, as well as the preventive measures mentioned above, two applications daily of the olive-oil-based formula, to the skin areas of the sacrum, hips and heels. This applications contained 0.33 ml in each spraying (one spraying for sacrum, one for hips and one for both heels).Any appearance of PU was recorded in the weekly follow-up report, stating also whether skin integrity was maintained or not. Any adverse effects occurring during the follow-up period were reported using a purpose-designed document.

#### Statistical Analysis

In order to achieve a power rate of 80%, to reject the null hypothesis (H₀: that a difference between the proportions of PU in both groups was below a non-inferiority limit of 10%), by means of ordinary asymptotic analysis, taking a level of statistical significance of 5%, and assuming that the proportion of PU in both groups was 45% [11], it was estimated that 612 patients would need to be recruited. Initially, we estimated a 15% withdrawal rate, but this was later raised to 25%, in view of the losses to follow up being experienced. Thus, a final sample size of 765 patients was required for a non-inferiority design where we stated a 10% absolute risk difference (from 0.1 to 10%) as the non inferiority margin (delta), to determine if the confidence interval of this absolute risk reduction included or not this delta value.

Continuous variables were summarised by mean values and the standard deviation, and the categorical variables were expressed in terms of absolute and relative frequencies. All variables were tested for normality using the Kolmogorov-Smirnov test. In order to evaluate the non-inferiority or otherwise of the olive oil treatment for the prevention of PU, absolute risk reduction (ARR) and relative risk (RR) values were estimated, and the chi square test applied, with confidence intervals of 95% in every case. In all cases, we examined whether the estimated delta value was within the confidence interval. Additionally, the T Student and the Mann-Whitney U tests were applied to determine differences in continuous variables by groups, and ANOVA was performed, with robust measures of central tendency in cases of heteroscedasticity (which was tested by the Levene test), by means of the Welch and Brown-Forsythe test. Kaplan-Meier curves were obtained and log-rank tests performed to determine the progress of the appearance of PU in both groups.

The non-inferiority analyses were carried out on both the per protocol and the intention to treat populations. The per protocol population included the subjects who completed the complete follow-up period and received the allocated target treatment, in order to force to the limit the conditions of difference between the treatments and to increase the null hypothesis rejection criteria. For the intention to treat analyses, multiple imputation was used to estimate missing data. This imputation process included five imputations for each variable estimated (incidence of any PU in the sacrum, hips or heels). SSPS 20 statistical software was used for all analyses.

## Results

**Fig 1:**
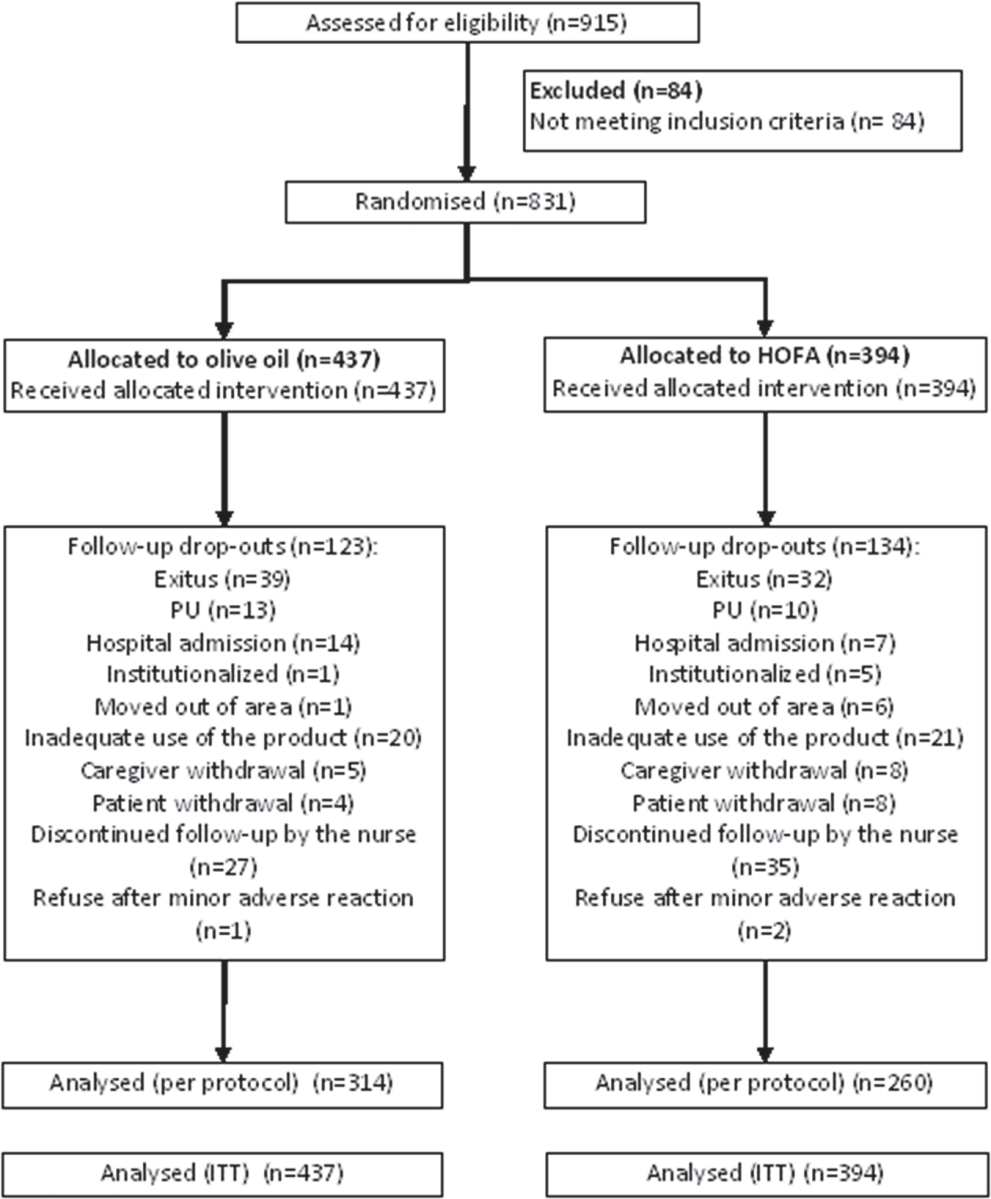
Patients Flow-chart.

The recruitment period lasted 17 months, from July 2012 to November 2013, and a total of 915 patients were recruited. Of these, 84 did not meet the inclusion criteria and were excluded, so that the final sample was made of 831 subjects. The remaining patients who did not complete the follow-up period are detailed in Fig 1.

*Table 1 Patients’ basal characteristics,* describes the baseline characteristics of the study groups. There were no differences between the groups.

**Table 1:** Patients’ basal characteristics.

		Group		
		**Olive oil (n = 437)**	**HOFA (n = 394)**	
		n (%) or $\bar{\text{X}}$ (SD)	n (%) or $\bar{\text{X}}$ (SD)	P
Sex	Male	123 (28)	105 (26.6)	0.641
		Female	314 (71.85)		289 (73.4)	
Level of mobility	Bed	160 (36.6)	149 (37.8)	0.246
		Armchair	212 (48.3)		172 (43.7)	
		Walk occasionally	65 (14.8)		73 (18.5)	
Antecedents of pressure ulcer		188 (43.0)	156 (39.7)	0.359
Cognitive impairment		299 (68.4)	284 (72.1)	0.256
Urinary or faecal incontinency		409 (93.6)	369 (93.7)	0.998
Diabetes		149 (34.1)	140 (35.5)	0.715
Other chronic diseases		383 (87.6)	354 (89.8)	0.326
Braden		12.97(2.30)	12.86(2.37)	0.490
MNA		6.98(2.09)	6.98 (2.07)	0.958
Technical aids		324 (74.1)	310 (78.7)	0.141
Heel protectors		141 (32.3)	119 (30.2)	0.549
Pressure relief cushion		169 (38.7)	140 (35.5)	0.351
Pressure relieving mattress		193 (44.2)	153 (38.8)	0.122
Equipment for mobilisation		44 (10.1)	44 (11.2)	0.652
Articulated bed		243 (55.6)	207 (52.5)	0.403
Used other product previously to prevent PU		104 (23.8	80 (20.3)	0.240

The average age of the caregivers was 55.70 years (SD 14.40) and the mean age of the patients was 80.56 years (SD 13.36). The mean level of PU risk, measured on the Braden scale, was 12.91 (SD 2.33), and the risk of malnutrition, assessed by MNA, was 6.98 (SD 2.08). Over half of the patients, in both groups (299; 68.4% and 284; 72.1% in the control and target groups, respectively), suffered some degree of some cognitive impairment, and in this respect there was no significant difference between the groups (p = 0.256).

The main treatment approaches used other than HOFA/olive oil were absorbent products for incontinence (olive oil group: 409(93.6%); HOFA group: 369 (93.7%); p = 0.998) and technical aids (olive oil group: 324(74.1%); HOFA group: 310(78.7%); p = 0.141), rather than protectors, pressure-relieving cushions or mattresses, articulated beds or lifting apparatus.

*Table 2 Basal skin integrity,* shows the distributions of the most frequently occurring types of injury, which mainly consisted of non-blanchable erythema, followed by partial thickness skin-loss.

**Table 2:** Basal skin integrity.

		Group		
		**Olive oil (n = 437)**	**HOFA (n = 394)**	
		**n (%)**	**n (%)**	p
Sacrum	Normal	413(94.5)	373 (94.7)	0.301
		**Non-blanchable erythema**	24(5.5)		19(4.8)	
		Partial thickness skin-loss	0 (0.0)		2 (0.5)	
Right heel	Normal	412(94.3)	380(96.4)	0.258
		**Non-blanchable erythema**	24(5.5)		14(3.6)	
		Partial thickness skin-loss	1(0.2)		0(0.0)	
Left heel	Normal	419(95.9)	384(97.5)	0.250
		**Non-blanchable erythema**	18(4.1)		10(2.5)	
		Partial thickness skin-loss	0(0.0)		0(0.0)	
Right trochanter	Normal	431(98.6))	389(98.7)	0.514
		**Non-blanchable erythema**	6(1.4)		4(1.0)	
		Partial thickness skin-loss	0(0.0)		1(0.3)	
Left trochanter	Normal	431(98.6)	391(99.2)	0.256
		**Non-blanchable erythema**	6(1.4)		2(0.5)	
		Partial thickness skin-loss	0(0.0)		1(0.3)	

Thus, the sacrum was affected in 45 cases (5.42%), the right heel in 39 (4.69%), the left heel in 28 (3.37%), the right trochanter in 11 (1.32%) and the left trochanter in 8 (0.96%). There were very few cases of partial thickness skin loss; in this respect, the most frequent site of occurrence was the sacrum, and only 2 subjects (0.35%) were affected. The intra-group analysis, showed significant reductions of tissue damage in the HOFA group, in sacrum (AAR 5.53; 95% CI: 1.79 to 9.27), right heel (AAR 3.79; 95% CI: 0.72 to 6.87), and left heel (AAR 2.89; 95% CI: 0.32 to 5.46), but not in right trochanter (AAR 0.65; 95% CI: -1.43 to 2.73), nor left trochanter (AAR 0.84; 95% CI: -0.6 to 2.28). In the olive oil group, the absolute risk reduction was significant in the five areas: Sacrum (7.07; 95% CI: 3.08 to 11.05), left heel (8.34; 95% CI: 4.55 to 12.13), right heel (5.97; 95% CI: 2.7 to 9.24), left trochanter (2.31; 95% CI: 0.48 to 4.13) and right trochanter (1.99; 95% CI: 0.06 to 3.92).

In the between-groups analysis, at the end of the follow-up period, in the per protocol analysis, none of the body areas evaluated presented risk differences for PU incidence that exceeded the 10% delta value set. The specific values in this respect for the sacrumwere: oliveoil, 8subjects (2.55%) vs HOFA, 8 (3.08%); ARR 0.53 (95%CI: -2.2 to 3.26), RR 0.83 (95%CI: 0.42 to 1.64).

Other areas analysed per protocol are detailed in *Table 3 Incidence of PU at the end of the follow-up and results of the per protocol and intention to treat analyses.*

**Table 3:** Incidence of PU at the end of the follow-up and results of the per protocol and intention to treat analyses.

PER PROTOCOL ANALYSIS				
	**HOFA (n = 260) n (%)**	**Olive oil (n = 314) n (%)**	**ARR (95% CI)**	**RR (95% CI)**
Sacrum	8 (3.08)	8 (2.55)	0,53 (-2,2 to 3,26)	0,83 (0,42 to 1,64)
Right heel	5 (1,92)	4 (1.27)	0.65 (-1.43 to 2.73)	0,66 (0,28 to 1,58)
Left heel	3 (1,15)	3 (0.96)	0.2 (-1.49 to 1.88)	0,83 (0,27 to 2,56)
Right trochanter	4 (1,54)	0 (0)	1.54 (0.04 to 3.03)	-
Left trochanter	1 (0,38)	1 (0,32)	0.07 (-0.91 to 1.04)	0.83 (0.12 to 5.87)
INTENTION TO TREAT ANALYSIS (imputed data)				
	**HOFA n (%)**	**Olive oil n (%)**	**ARR (95% CI)**	**RR (95% CI)**
Sacrum	9 (2.28)	11 (2.52)	-0.23 (-2.31 to 1.85)	1,1 (0.58 to 2.1)
Right heel	137 (34.77)	125 (28.6)	6.17 (-0.16 to 12.5)	0.82 (0.72 to 0.94)
Left heel	135 (34.26)	124 (28.38)	5.89 (-0.42 to 12.2)	0.83 (0.72 to 0.95)
Right trochanter	136 (34.52)	121 (27.69)	6.83 (0.53 to 13.12)	0.8 (0.7 to 0.92)
Left trochanter	55 (13.96)	47 (10.76)	3.2 (-1.28 to 7.69)	0.77 (0.6 to 0.98)

AAR: Absolute risk reduction; RR: Relative risk

The intention to treat analysis showed that the lower limit of the confidence interval established for non-inferiority was not exceeded in any of the areas assessed.

**Fig 2:**
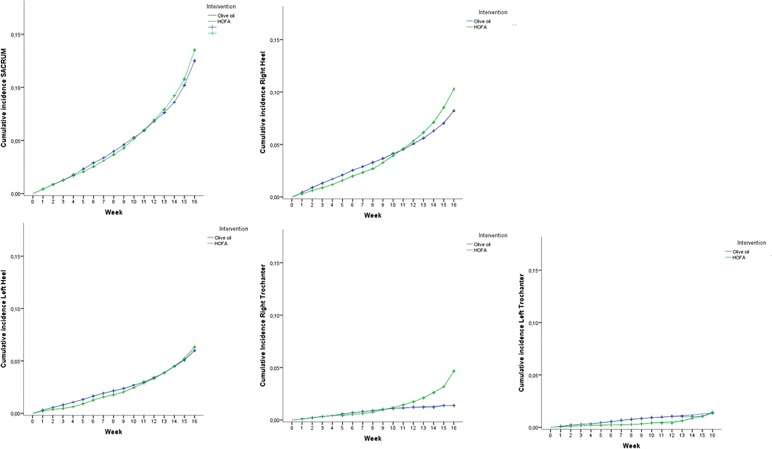
Evolution of incidence of PU during the 16 weeks.

**Fig 3:**
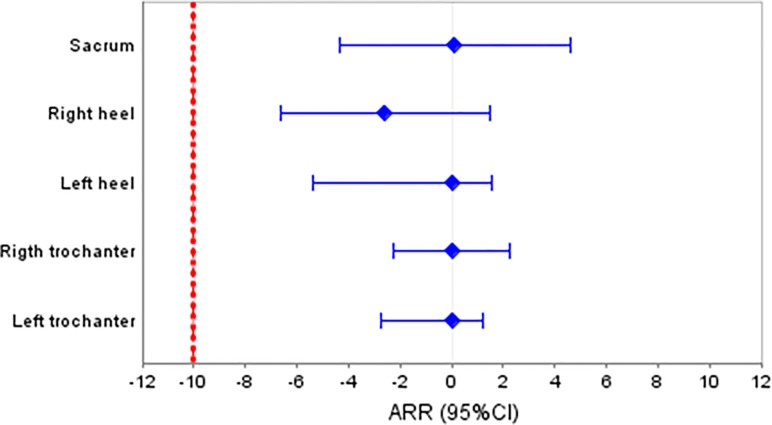
AAR of PU at week 16, and confidence intervals, with the non-inferiority margin (red line).

*Fig 2* shows the evolution of PU incidence during the 16 weeks, and Fig 3 shows the distribution of the confidence intervals for each skin area (the red line indicates the limit of non-inferiority).

A subgroup analysis was carried out to compare patients who did not comply with the full follow-up and those who did, with the aim of identifying the possible reasons for their withdrawal from the trial, before accepting or rejecting the null hypothesis. For this analysis, age, sex, level of physical activity, prior use of another product to prevent PU, cognitive status, presence of comorbidities or incontinence and availability of technical devices for the prevention of PU were taken into account. No significant differences were found in any case.

With respect to adverse effects observed during the study, two cases occurred in patients using HOFA and one in a patient using olive oil (itching or redness). In neither of these two groups was the effect severe, and it resolved spontaneously; nevertheless, these patients chose to leave the study.

## Discussion

The aim of this trial was to assess whether the use of a formula of extra virgin olive oil to prevent the appearance of PU was inferior to the use of HOFA for this purpose, for high-risk immobilised home-care patients. The results obtained confirm the non-inferiority of the olive oil treatment; there were no differences exceeding the lower limit of the confidence interval and thus olive oil therapy achieves an effective prevention of PU in these circumstances.

Previous studies of the use of HOFA did not continue follow up beyond one month, with maximum durations of seven days [25], nine days [24] and thirty days [21]. In the present study, the follow up period was 112 days (16 weeks). This extended period was of considerable clinical significance, since in the context of home care, interventions to prevent PU should be focused on achieving a lasting effect, since the risk conditions of these patients are very unlikely to change.

The characteristics of the patients and family caregivers enrolled in this study are very similar to those of the population of the home care population in Spain [29, 30] and in Europe [31, 32]—elderly, at high risk of developing PU, cognitive impairment and multiple pathologies—since the data were compiled in the course of routine clinical practice by nursing personnel. The data are also similar to those for the subjects included in studies performed to demonstrate the effectiveness of HOFA, although these took place in different environments, such as hospitals or nursing homes [21, 24, 25].*Table 1 Patients’ basal characteristics*.

Among the caregivers, the data are comparable with those published following the study conducted in Spain by Imserso in 2004, according to which 83.6% of caregivers were women. They are also similar to the results obtained in neighbouring countries (Germany, Greece, Italy, Poland, Sweden, United Kingdom), according to the EUROFAMCARE study, in which the proportion of female caregivers ranged from 60% to 85% [33]. The mean age of the caregivers in our study was 55.7 years (SD 14.40). It is noteworthy that they are not young, but 83.4% are informal caregivers and 64.4% are sons or daughters of the patients, which accounts for their age. *Table 1 Patients’ basal characteristics*.

Among the patients included in the study, the mean age and sex are similar to those in a study comparing the effectiveness of two HOFA in the treatment of Stage I PU in hospitalised geriatric patients [24]. Most of the patients, in both groups, were at high risk of developing PU. For the present study, the subjects with this risk profile were selected in order to test whether the intervention was effective in clinical practice in the home environment. This hypothesis was confirmed, even in the relatively adverse context established. Home care patients are very fragile and dependent; they present many risk factors and require increasingly complex treatment. The goal of the primary care team is to respond to the patient’s wish to remain at home for as long as possible, under the best possible conditions. If the development of PU can be prevented, this will increase these patients’ quality of life and reduce morbidity and mortality, as well as the consumption of materials and human resources. A noteworthy outcome of this study is that it obtained equivalent results in a complex population, ensured by the inclusion criteria applied and the resulting homogeneity of the groups. *Table 2 Basal skin integrity.*

Many of the patients included were at risk of malnutrition, as reflected by the mean MNA value of less than 7. Our literature search did not reveal any study of HOFA that specifically included this parameter, despite evidence of the influence of nutrition on the development of PU reported in various studies and clinical practice guidelines [19, 34, 35]. We decided to include MNA scale because Braden scale measured is general and subjective. Malnutrition is a major risk factor and to ensure the inclusion criteria, needed to be comprehensive in measuring the nutritional status with a specific and validated scale, in this case MNA scale.

Regarding physical activity, almost half of the patients in both groups spent most of their time in bed or seated, and were thus subject to forces of pressure, friction and shear, placing them at high risk of developing PU. A study comparing the effectiveness of two different HOFA in preventing Stage 1 PU reported similar results in this respect, with 69.4% and 75% of patients restricted to a prone and/or seated posture [24].

Over half of the patients, in both groups, suffered cognitive impairment, and made high use of absorbent products for incontinence; both are risk factors, which in addition to the others mentioned above, predispose these subjects to the development of ulcers [35].

Only one adverse effect (and this was slight) was recorded among the olive oil treatment group, and so the product is considered safe for topical application to intact skin. It was only possible to compare these results with HOFA studies conducted in hospitals or nursing homes, because our literature search did not obtain any randomised clinical equivalence trials studying the use of olive oil to prevent PU in the home care environment.

With respect to external validity and applicability in clinical practice, the results of this study support the widespread use of the olive oil formula (non-oxygenated fatty acids) in the prevention of PU in primary care, and by extension in hospitals, as the patients studied are very similar to those treated in hospitals and nursing homes. It should be stressed that the cost of treating PU represents a major outlay for the health system and for society in general and also increases the burden of care on caregivers, requiring considerable time, materials and resources. The lower cost of the product makes it more accessible to the population in general and to the healthcare system, and offers a viable alternative to standard HOFA treatment, promising a significant reduction in pharmaceutical expenditure and alleviating the burdens placed on health care personnel and caregivers, by reducing the incidence of PU. *Table 3 Incidence of PU at the end of the follow-up and results of the per protocol and intention to treat analyses.*

Further studies should be conducted to investigate the action mechanism by which olive oil contributes to preventing PU and to determine the pathogenesis of PU, with respect to factors such as friction and shear forces, capillary circulation and microvascular, histological and molecular changes.

### Limitations

Analysis of the study sample losses to follow up showed there were no significant differences in this respect between the groups, and so they are unlikely to be caused by the characteristics of the intervention. The mode of application of the product is very simple, but to avoid variability in the application (and thus potential bias)—a possibility under real conditions due to the high number of participants, with no specific instruction in the use of the product—the nurses collaborating with the study gave the caregivers training in this respect. Moreover, all were given an instruction sheet and an explanatory video, thus ensuring consistent use of the product by all participants. Conditions were also controlled by masking the product and randomising its use among the technical personnel involved.

To avoid the heterogeneity within the groups and the risk of obtaining a very low or zero incidence of PU, patients at low risk of developing this condition were excluded. In our opinion, the effect of the new product on this population would improve their skin quality, but we would be unable to determine whether the low incidence of PU was the result of application of the product, or of the absence of risk. This control of the internal validity of the study is a limitation as regards applicability of the product among low-risk patients, as none were included in the study.

Patients who were admitted to hospital during the follow-up period were excluded from the study. This was done in order to avoid contamination regarding application of the product, because when such a situation occurs, physicians could apply the products available in the hospital, which would probably be different from those assigned according to the study protocol. Further studies are needed to consider the effectiveness of this product in acutely hospitalised patients, because although such patients are similar to those included in the HOFA studies, it is necessary to clarify whether the caregiving environment and the specific care provided influences the outcome.

We used the Braden Scale for assessment of the risk for skin break down and the Mini Nutritional Assessment for assessment of nutritional status. But we only measure these ones at the beginning of our study, because we believe that patients studied did not change significantly the results of these scales in only 16 weeks.

## Supporting Information

S1 Protocol Protocol Translate. (PDF)

S1 CONSORT Checklist CONSORT Checklist. (PDF)

S1 Informed Consent Informed Consent. (PDF)
