# Supplementary material for: Topical Olive Oil Is Not Inferior to Hyperoxygenated Fatty Aids to Prevent Pressure Ulcers in High-Risk Immobilised Patients in Home Care. Results of a Multicentre Randomised Triple-Blind Controlled Non-Inferiority Trial
Source: PLoS One. 2015 Apr 17;10(4):e0122238. doi: 10.1371/journal.pone.0122238 (PMC4401455; doi:10.1371/journal.pone.0122238)
Supplement: S1 Protocol — (PDF) [file pone.0122238.s001.pdf]

TITLE:

Olive-oil effectiveness in the prevention of pressure ulcers caused in immobilized patients within the scope of primary health care: study protocol for a randomised controlled trial

ABSTRACT

Backgrounds

Pressure ulcers (PU) are considered as an important issue affecting mainly to immobilized elderly patients, thus, increasing the care burden to the professional staff of the healthcare service as well as the pharmaceutical expenditure. There are a number of studies on effectiveness of different products used for the prevention of UP, however, most of these studies were carried out within the hospital scope using basically hyperoxygenated fatty acids (HOFA). There are no studies focused specifically in the use of olive oil-made products and therefore the research group is intended to seek the most cost-effective treatment and achieving an alternative treatment.

Methods and Design

Main objective: To assess the effectiveness of the use of olive oil comparing it with the use of HOFA for immobilized patients at their home at risk of suffering pressure ulcers (PU).

Secondary objective: To assess the cost-effectiveness balance of this new application in regards to the HOFA.

Design: Non-inferiority, triple-blinded, parallel, multi-centre, randomized clinical-trial.

Scope: Population attending Primary Health Centres in Andalucia (Spain) in the regional areas of Malaga, Granada, Seville and Cadiz.

Target population: immobilized patients at risk of suffering PU.

Procedure: Application of an olive-oil based magistral formula to the target group and applying HOFA to the control group. Following-up period 16 weeks.

Main variable: Pressure ulcers in the patient. Secondary variables: Socio-demographic and clinical information, caregiver information and whether there is technical support in place.

Statistics Analysis: It shall be applied the Kolmogorov-Smirnof test, symmetry and kurtosis analysis, bivariate analysis using the Student's-T and Chi-Squared tests as well as the Wilcoxon and the Man-Whitney U tests, ANOVA and multivariate logistic regression analysis.

Discussion: The usual use of olive-oil based formulas shall be effective to prevent the PU in immobilized patients leading to a more cost-effective product and alternative treatment.

Trial registration:

Clinicaltrials.gov Identifier: NCT01595347.

KEY WORDS

Pressure ulcers/Prevention/ Olive-oil/Hyperoxygenated fatty acids

BACKGROUND

The PU are considered as an economic, social and health problem which does not just decreases the quality of life of the patients and their social and familiar environment but it also involves the worsening of the patient's prognosis as well as it decreases the life expectancy of those patients suffering PU due to the high number of related physical complications. A pressure ulcer (PU) might be defined as injuries to the skin caused by ischemia process which might affect and necrotising those areas in the epidermis, dermis, subcutaneous tissues and muscles where the PU appears, which may also affect bones and joints in the most severe cases. PUs tend to appear when the soft tissues are compressed between two planes, namely, the bony prominences of the patient and an external surface (1) jointly with the vascular occlusion produced by the external pressure and the endothelial damage for arterioles and microcirculation mainly due to the tangential strength shear and frictional forces. The PU involves the alteration of a basic need for patients as it is to preserve the skin integrity. Pressure ulcers may appear in any part of the body, being the bony prominences (sacrum, hips, heels) the most common, affecting mainly elderly people, immobilized patients suffering severe acute disease and patients with neurological deficits.

The PU incidence and prevalence are some of the most representative indicators of the quality of Nursing care. The data on PU prevalence in Spanish hospitals (8.24%) (2) are very similar to its neighbouring countries as Italy (8.3%), France (8.9%), Germany (10.2%) or Portugal (12.5%)(3)(4)or further countries as Jordan (12%) (5). The highest records of prevalence are registered in Ireland (18.5%), Wales (26.7%)(6)Belgium (21.1%), United Kingdom (21.9%),

Denmark (22.7%) or Sweden (23.0%)(7). The appearance of PU's in hospitalised patients is a frequent complication which involve a negative impact for the patient's health and it often causes the extension of the hospitalization period and thus, the increase in the health care expenses.

In regards to the nursing care at home, there are no specific records which might compare the prevalence in patients included in nursing care at home programs given that the characteristics of the health systems vary considerably from one country to another. In nursing homes, the most important study carried out might be the study recently published by Park-Lee(8), which represents the overall of American elderly care homes which evidence a PU's prevalence at (11%), similar to the Spanish rate.

In regards to the scope of the Primary Health Centres, according to the data achieved from the second National Study on Pressure Ulcers Prevalence in Spain, in 2005 (9), the crude prevalence rate of the PU was registered at 3.73% and the mean prevalence rate was registered at 9.11% ± 10.9% for those patients over 14 years which are included in the nursing care at home service. The most common appearance areas are sacrum and heels, affecting mainly to the population group including people over 65 years.

Pressure ulcers are considered nowadays as an important health problem involving a financial impact as it increases substantially the pharmaceutical expenditure. The total spending derived from the treatment of the PU reaches 5% of the annual health spending (10), thus involving a heavier health care workload for the professional staff.

The main risk factors causing the PU are: immobility, incontinence, malnutrition and awareness level; PU are an indirect indicator of the health care quality, thus, a low rate of PU in patients evidence high-quality health care services based on preventive measures. The assessment of risk factors, the use of support surfaces, repositioning of the patient, a good nutritional status and the skin moisture or the use of the checking lists are adequate strategies to prevent the PU (11)(12)(13)(14)(15)(16)(17) (18) which have evidenced their effectiveness even in elderly patients (19).

There are a number of products intended for the prevention of the PU such as oil compounds based on Hyperoxygenated fatty acids (HOFA), on which a high number of studies have proven its effectiveness by maintaining the skin integrity avoiding the PU or by postponing the

appearance of PU (20)(21)(22)(23)(24)(25). However, one inconvenience of this treatment is its high cost for long-term treatments.

In the Mediterranean culture, the olive-oil is an essential element with proven effects as basic element of the diet in the prevention of cardiovascular diseases, however, jointly with its nutritional properties; it also provides beneficial effects when topically applied, mainly due to its moisturising and emollient qualities, which have been used as unguent due to its composition. The olive oil is composed of triglyceride at 98%, including predominantly monounsaturated oleic acids, which due to its anti-inflammatory properties has been proven to be essential for the health and the skin maintenance as such properties are similar to the ibuprofen (which are attributable to Oleocanthal according to recent studies) which may accelerate the recovery and healing process (26). Furthermore, due to the high rate of polyphenols, natural antioxidants included in the Extra Virgin Olive Oil (EVOO), its use mitigates the inflammatory process thus explaining its beneficial effects for the skin inflammatory disorders (27)(28). The role of the oleic acid is a key feature within the reconstruction of the cell membranes, providing higher smoothness to the dermis by restoring the skin humidity levels, thus moisturising the skin and providing it with elasticity. Besides, the oil components such as the phenolic compounds and chlorophyll, have a high antioxidant power and therefore, anti-aging effects, apart from accelerating the dermis healing process. Besides, it shall be also mentioned that E vitamins are included in the oil composition, which is an excellent source for protection against the free radicals causing the cell oxidation (29). The excess of free radicals in the organism accelerate the aging and as a result, the membranes of the epithelial cells are modified, thus making difficult the skin nutrition, damaging also the collagen and elastin fibres which make the skin to lose firmness and elasticity. The free radicals are a 7-electron oxygen atom (the oxygen stable atom has 8 electrons and becomes unstable when it loses one electron). When such electron is missing, it borrows it from the cell membrane and produces another free radical, causing a chain reaction which is fought with the anti-oxidants action which neutralizes the oxygen atoms. Among the exogenous antioxidants acting in the membrane lipids, it is included the E-vitamins, carotene, polyphenols and flavonoids, which makes the EVOO a natural source of such components (30).

The main role of the oil is its anti-oxidant function and for such purpose, the oil shall have a low rate of peroxides and a high rate of polyphenols. The extra virgin olive oil includes 330-500 mg of polyphenols per fat kg and less than 20 milliequivalent weight of peroxide per fat kg (the

composition of the hyperoxygenated fatty acids includes from 40-50 mg of polyphenols per fat kg and from 230-340 milliequivalents of peroxide per fat kg). Due to its lipid composition, it is completely compatible with the cells of human tissues, thus involving that its topic use does not cause allergy, neither irritation. Such properties may lead us to consider the possible protective effect on the appearance of PU, which are similar to the effects of HOFA, but for a much lower cost, apart from offering an alternative treatment. Furthermore, there are no previous experimentations with this products applied for the prevention of PU in nursing care at home. The main aim of this trial is to assess whether the effectiveness of the olive oil use is lower or higher than the HOFA in the prevention of PU in immobilized patients within the nursing care at home and; as a secondary aim, to assess the cost-effectiveness of this new intervention in regards to the use of HOFA.

METHODS/DESIGN

The design of this study is a non-inferiority, triple-blinded, parallel, multi-centre, randomized clinical-trial. The hypothesis of this study is that the difference in the appearance of PU in immobilised patients at home applying olive oil is lower than 10% face to those patients applying HOFA.

Two procedures shall be performed: usual care and applying HOFA to the control group and usual care and applying an olive-oil composition to the target group. The main outcome shall be the appearance of II-stage PU. The accuracy of the trial in regards to the control medicine (HOFA) is widely supported due to the historical favourable results of such medicine face to the placebo (25) as per recommendations of the International Conference of Harmonization and just because the efficiency conditions of the mentioned medicine shall remain intact for the purpose of this trial.

The target population shall gather patients included in the immobilised-patients program within the nursing care at home service provided in the Health Centres in Andalucia (Spain), and more specifically in those regions of Malaga, Granada, Seville and Cadiz.

The inclusion criteria established shall be: patients over 18 years; patients supported by a family or a remunerated caregiver for the application of the treatment; diagnostic issued by a nurse as “Risk for Impaired Skin Integrity”, by applying the Braden Scale for prediction of PU risk, thus, such patients shall report a high (score <= 12) or moderated risk (score 13-16), and;

patients with nutritional status assessed according to the Mini Nutritional Assessment (MNA) scoring 10 or lower.

In regards to the exclusion criteria, it has been established: patient rejection to take part in the trial; patients with a usual address different than the area of the health centre where the study is carried out, or plans to be out of the usual address during the follow-up period; those patients which have been hospitalized during the sampling period, terminally-ill patients or those who already have PU.

All the patients included in the trial shall receive the information concerning the aim of the trial in writing which shall be duly signed by the researchers. The patients shall confirm their participation in the trial and sign the informed consent. In those cases when the patients have cognitive impairment, it shall be the legal guardian who shall sign the informed consent. The patients who do not confirm their consent shall be substituted by other patients selected by the same procedure of general randomised selection, thus, extending the selection process as long as necessary.

Sample selection

The Directors and care service coordinators of each Health Centre shall be duly informed about the trial, and they shall be asked for their availability as well as their consent to take part in the trial. The nursing staff of each health centre shall take part in this trial freely, being in such case responsible for the research study implementation and follow-up in their respective Health Centres.

The patients shall be assessed to determine whether they comply with the inclusion requirements by checking the records on patients included in the nursing at home program for immobilized patients. At this point, such patients shall sign the informed consent and following, their details shall be randomized by a computer system beyond the control of the professional staff which shall be processed at the clinical trial control centre.

In order to achieve a power rate of 80.00% to reject the null hypothesis (H_0: the difference between proportions p1 and p2 is lower than the non-inferiority limit), by means of an ordinary asymptotic analysis estimating (non-inferiority) unilateral proportions for two independent series, taking into account that the level of statistical significance is 5.00% and assuming that: the proportion in the Reference control Group is 45.00%; the proportion in the Experimental Group is 45.00% (9); the proportion of experimental units in the Reference

Group is 50% related to total and the Non-inferiority limit (delta) is 10.00%, then, it shall be necessary to include 306 experimental units in the Reference Group and 306 units in the Experimental Group, totalising 612 units in the study. Taking into account that the expected withdrawal rate is 15.00%, it shall be necessary to enrol 360 experimental units in the Reference Group and 360 units in the Experimental Group, totalising 720 experimental units in the trial.

The main outcome shall be the incidence of II-stage PU in patients during 16-weeks' follow-up. The appearance of II-stage PU shall be confirmed by the inspection of those areas where the product has been applied (sacrum, hips and heels).

As secondary end-points, it shall be applied cost measures which shall allow us to know the cost-effectiveness balance of the new product (olive-oil formula) in regards to the HOFA-based products. As inputs of such model, it shall be used the cost for each treatment, defined as the number of units (NE_{ij}) necessary to carry out a 16-weeks treatment (112 days) multiplied by the price of 1 one unit (P_i and P_j) (the price of each unit shall be achieved from the information provided by each supplier according to the market price). The number of units shall be the resulting number when dividing the treatment period (DT_{ij}) (112 days) by the number of days (d_{ij}) it does least the unit used and duly specified according to the trial protocol on each treatment arm. The resulting amount achieves shall be rounded up, despite the total unit has not been fully used: $NE_{ij}= DT_{ij}/d_{ij}$.

As variable cost, it shall be established according to the number of visits of the nurse to the patient's home during the trial period (VE_i), which shall act as proxy variables on the level of care required. The mean time spend in each visit shall be estimated according to the standard value of time within the community health nurse working hours spend during a nursing care-at-home visit (this information shall be achieved from the Human Resources Management Division of the different Health Districts and it shall be carried out an assessment test on the length of 30 visits to patients with the characteristics complying with the inclusion criteria, randomly selected among the health centres included in the study, prior to the commencement of the trial). Structural fixed costs shall not be included as in all cases the care assistance is provided at the patient's home and the services are already included in the aforementioned variable. It shall not be possible to assess the cost derived from the family care, since it is quite difficult to achieve and has a high variability, therefore, this shall not be

included in the model. In those cases when there is a remunerated caregiver, such cost shall not be included in the total costs either, as it would create a bias in the estimation when comparing them with those patients who are not assisted by a remunerated caregiver. Furthermore, those intangible costs derived from certain aspects as it is the pain level derived from the process shall not be neither included. Therefore, the estimation of total costs (CT) in the model shall remain as it follows: $CT_{ij}=NE_{ij}+VE_{ij.}$

The appearance of PU shall be considered as the effectiveness variable. It shall be taken into account a finite number of states: appearance of PU (U_1) and non-appearance of PU (U_0). The incidence shall be estimated in percentages which shall be used for the incremental estimations on cost-effectiveness.

Furthermore, variables on characterization of patients and caregivers shall be included to assess socio-demographical data such as the time registered in the care service for immobilized patients, information about previous cases of PU, comorbidity, nutritional and cognitive status, moisture (incontinence) apart from taking into account the ulcer location as well as the availability of technical support items (mattress and cushions to avoid bedsores, adjustable beds, etc.). In regards to the caretaker, it shall be taken into account the age, gender and whether he/she is a family or remunerated caregiver.

A basal-assessment shall be performed on all patients at the beginning of the trial and this shall be repeated each 7 days up to the conclusion of the follow-up period, or until the PU appear in patients. All the information concerning the application of the products shall be registered in the weekly evaluations.

<u>Procedure</u>

The new procedure consists on the application of a magistral formula available whether in liquid and spray, which contains 95% EVOO. This product shall have the same appearance than the hyperoxygenated fatty acid, thus, using the same presentation which shall be just solely used by patients and nurses. This intervention shall be applied to both groups (control and target group). Both, the patients of the control group as well as those patients part of the target group, shall receive the preventive instructions which are stated in the Clinical Practice Guidelines on deterioration of skin integrity issued by the Health District Malaga. For such purposes, the caregivers of both groups shall be duly trained on the mentioned procedures. Jointly with these preventive measures, the patients included in the control group shall receive

two applications per day of the HOFA-based product in the skin, more specifically, in the sacral area, the hips and heels. For the purpose of this trial, we shall use a HOFA product bearing the CE marking, IIb Class, with a higher classification that the rest of products existing in the market. The product is to be applied topically and includes hyperoxygenated fatty acids, Equisetum Arvense, Hypericum Perforatum and perfume. On their part, the target group shall receive, jointly with the preventive measures mentioned above, two daily applications of the magistral olive-oil-based formula in the skin areas of sacrum, hips and heels. The appearance of PU shall be registered in the weekly follow-up report in which it shall be included whether the skin integrity is maintained or not. In the event that during the follow-up period there is any adverse effect, the researching group shall inform accordingly and such event shall be notified by means of the specific document designed for such purpose.

Statistical Analysis

Initially, it shall be performed a descriptive analysis of the variables included in the trial. Continuous variables shall be summarised with means, standard deviation, median, and interquartile range. The categorical variables shall be shown in absolute and relatives frequencies. It shall be performed an analysis on the normal distribution by the Kolmogorov-Smirnov test, symmetry analysis and kurtosis.

With the aim of assessing the non-inferiority of the olive-oil for the prevention of PU, it shall be carried out a contrast of association between the appearance of PU and the kind of treatment received (olive-oil-based cream/conventional treatment), by absolute and relative measures of effect sizes (complete reduction of risk, number needed to treat-NNT-, Relative risk and relative reduction of risk), with their respective confidence intervals at 95%. It shall be determined whether the confidence interval includes or not the estimated delta value from sample, in order to reject the null hypothesis or not.

It shall be carried out a bivariate analysis using the Student's-T and Chi-Squared tests according to the characteristics of the variables analysed in the event that these are normally distributed. Otherwise, it shall be applied non-parametric tests, as the Wilcoxon and Man-Whitney U tests. Likewise, it shall be applied ANOVA for the association of quantitative and qualitative variables in the necessary cases, as well as robust measures of central tendency in cases of homoscedasticity (which shall be tested by the Levene test) by means of the Welch and Brown-Forsythe test.

It shall be carried out analysis according to the Kaplan-Meier curves and log-rank test to determine the progress of the appearance of PU in both groups, taking into account the treatment applied, the gender, age, the nutritional status of the patients, the functional degree and the caregiver's age.

For those analysis on cost-effectiveness, it shall be achieved the effectiveness measures previously established (U_1 y U_0). It shall be estimated the increase of the costs for each option (ΔC_{ij}), the effectiveness rate of both options, the ratio cost and efficiency of each treatment and the incremental cost-effectiveness ratio (ICER) of each option. This value shall enable us to assess the necessary cost to increase by 1% the possibilities of success when preventing PU in these patients by using each product.

The analysis shall be carried out according to the protocol, as it is established for the non-inferiority studies, for the purpose of forcing to the limits the conditions of difference between the treatments and increasing the null hypothesis rejection criteria. Nevertheless, it shall be carried out an analysis which shall be intended to compare both analysis and assessing, in those cases of non-coincidence, the sub-groups of patients who did not complied with the trial protocol, with the aim of identifying the possible reasons for the withdrawal from the trial, before accepting or rejecting the null hypothesis. The confidence intervals of the main result variable shall be analysed to determine whether the difference found is within the limit established for the delta value. The statistics analysis shall be performed by the SSPS 20 software.

Ethical and Legal Issues

The Ethics and Research Committee of Malaga (Northwest District) granted the approval for this trial. The regulations on Good Clinical Practice shall be observed at any time, as well as the ethical principles established for the research on human beings pursuant to the Declaration of Helsinki and further amendments thereto. The clinical information shall remain biased from the identification details and the databases shall be coded and stored in specific computers solely intended for the project. All the registries shall be carried out observing all the dispositions pursuant to the legislation in force in regards to personal data protection according to the Act 15/1999 dated December 13th, as well as on safety of computerised files which may store personal data information, and more specifically, in regards to the access through communication nets (Royal Decree 994/1999 dated June 11th) and access to confidential information for scientific purposes pursuant to the Commission Regulation CE No.

831/2002 by the European Union and Act 41/2002 dated November 14th which is considered as the legal standards on Patient's Autonomy and Rights and Duties in terms of Clinical Information and Documentation.

The staff responsible for such purpose shall manage the data pursuant to the instructions provided by the person responsible of the treatment. Such data shall not be applied or used for a different purpose than those stated in the respective authorization; neither shall be reproduced to other people even thought if it is for registration purposes. Once the aim of the trial has been carried out, the personal data collected shall be destructed, wiped or given back to the person responsible of the treatment as well as any other supporting means or documents in which there is any personal data related to the project. Every patient shall be verbal and written informed about the objectives of the project and its methodology. Each individual shall sign the respective informed consent form.

TRIAL STATUS

Patient inclusion in this trial started on 1 November 2012 and is scheduled to continue until 31 July 2013.

LIST OF ABBREVIATIONS USED

PU: Pressure Ulcers

HOFA: Hyperoxygenated fatty acrids

GNEAUPP (*as per its Spanish acronyms*): National Committee for Research and Counselling on Pressure Ulcers and chronic wounds.

MNA: Mini Nutritional Assessment Scale

EVOO: Extra Virgin Olive Oil

CONFLICT OF INTEREST STATEMENT

The authors declare there is no conflict of interest.

REFERENCES

1. Hanson D, Langemo DK, Anderson J, Thompson P, Hunter S. Friction and shear considerations in pressure ulcer development. Adv Skin Wound Care. 2010 Jan;23(1):21–4.

2. Soldevilla Agreda JJ et al. 3.er Estudio Nacional de Prevalencia de Úlceras por Presión en España, 2009: Epidemiología y variables definitorias de las lesiones y pacientes. Gerokomos. 2011;22(2):77 –90.

3. Kottner J, Lahmann N, Dassen T. [Pressure ulcer prevalence: comparison between nursing homes and hospitals]. Pflege Z. 2010 Apr;63(4):228–31.

4. Wilborn D, Halfens R, Dassen T, Tannen A. [Pressure ulcer prevalence in German nursing homes and hospitals: what role does the National Nursing Expert Standard Prevention of Pressure Ulcer play?]. Gesundheitswesen. 2010 Apr;72(4):240–5.

5. Tubaishat A, Anthony D, Saleh M. Pressure ulcers in Jordan: a point prevalence study. J Tissue Viability. 2011 Feb;20(1):14–9.

6. James J, Evans JA, Young T, Clark M. Pressure ulcer prevalence across Welsh orthopaedic units and community hospitals: surveys based on the European Pressure Ulcer Advisory Panel minimum data set. Int Wound J. 2010 Jun;7(3):147–52.

7. Vanderwee K, Clark M, Dealey C, Gunningberg L, Defloor T. Pressure ulcer prevalence in Europe: a pilot study. J Eval Clin Pract. 2007 Apr;13(2):227–35.

8. Park-Lee E, Caffrey C. Pressure ulcers among nursing home residents: United States, 2004. NCHS Data Brief. 2009 Feb;(14):1–8.

9. Agreda JJS, Soriano JV, Santos JMM, Casanova PL, López JR, Bou J-ET i, et al. 2º Estudio Nacional de Prevalencia de Ulceras por Presión en España, 2005: epidemiología y variables definitorias de las lesiones y pacientes. Gerokomos: Revista de la Sociedad Española de Enfermería Geriátrica y Gerontológica. 2006;17(3):154–72.

10. Javier SA, José, Joan-Enric T i B, John P, José VS, Lorena SM, M MS, José. Una aproximación al impacto del coste económico del tratamiento de las úlceras por presión en España. Gerokomos: Revista de la Sociedad Española de Enfermería Geriátrica y Gerontológica. 2007;18(4):201–10.

11. Reddy M, Gill SS, Rochon PA. Preventing pressure ulcers: a systematic review. JAMA. 2006 Aug 23;296(8):974–84.

12. Delmore B, Lebovits S, Baldock P, Suggs B, Ayello EA. Pressure ulcer prevention program: a journey. J Wound Ostomy Continence Nurs. 2011 Oct;38(5):505–13.

13. Salcido R, Lee A, Ahn C. Heel pressure ulcers: purple heel and deep tissue injury. Adv Skin Wound Care. 2011 Aug;24(8):374–380; quiz 381–382.

14. Walker Sewill DK, Van Sell S, Kindred C. Pressure ulcer prevention: utilizing unlicensed assistive personnel. Crit Care Nurs Q. 2010 Dec;33(4):348–55.

15. White-Chu EF, Flock P, Struck B, Aronson L. Pressure ulcers in long-term care. Clin. Geriatr. Med. 2011 May;27(2):241–58.

16. Dealey C. Skin care and pressure ulcers. Adv Skin Wound Care. 2009;22(9):421.

17. Lee T-T, Lin K-C, Mills ME, Kuo Y-H. Factors related to the prevention and management of pressure ulcers. Comput Inform Nurs. 2012 Sep;30(9):489–95.

18. Jaul E. Assessment and management of pressure ulcers in the elderly: current strategies. Drugs Aging. 2010 Apr 1;27(4):311–25.

19. Gorecki C, Brown JM, Nelson EA, Briggs M, Schoonhoven L, Dealey C, et al. Impact of pressure ulcers on quality of life in older patients: a systematic review. J Am Geriatr Soc. 2009 Jul;57(7):1175–83.

20. Martínez Cuervo F and PG. La efectividad de los ácidos grasos hiperoxigenados en el cuidado de la piel perilesional, la prevención de las úlceras por presión, vasculares y de pie diabético. Gerokomos. 2009;20(1):41 –46.

21. C F, E, Gallart. Estudio experimental para comprobar la efectividad de los ácidos grasos hiperoxigenados en la prevención de las úlceras por presión en pacientes ingresados. Enfermería clínica. 2001;11(5):179–83.

22. Torra i Bou J-E, Rueda López J, Segovia Gómez T, Bermejo Martínez M. [Topical administration of an hyperoxygenated fatty acid compound. Preventive and curative effects on pressure ulcer]. Rev Enferm. 2003 Jan;26(1):54–61.

23. Segovia Gómez T, Bermejo Martínez M, Molina Silva R, Rueda López J, Torra i Bou JE. [Skin care and pressure ulcer. Hyperoxygenated fatty acids in the prevention of pressure ulcers and treatment of stage I lesions]. Rev Enferm. 2001 Sep;24(9):18–22.

24. Candela-Zamora MD, Martín-Gómez MA, Solas-Gómez B, Fernández-Pérez C, Martín-González M, Manzanedo-Basilio L, et al. [Comparative study of the effectiveness two of hyperoxygenated fatty acids in the treatment of grade I ulcers in geriatric hospitalized patients]. Enferm Clin. 2010 Feb;20(1):10–6.

25. Torra i Bou JE, Segovia Gómez T, Verdú Soriano J, Nolasco Bonmatí A, Rueda López J, Arboix i Perejamo M. The effectiveness of a hyperoxygenated fatty acid compound in preventing pressure ulcers. J Wound Care. 2005 Mar;14(3):117–21.

26. Beauchamp GK, Keast RSJ, Morel D, Lin J, Pika J, Han Q, et al. Phytochemistry: ibuprofen-like activity in extra-virgin olive oil. Nature. 2005 Sep 1;437(7055):45–6.

27. Wahle KWJ, Caruso D, Ochoa JJ, Quiles JL. Olive oil and modulation of cell signaling in disease prevention. Lipids. 2004 Dec;39(12):1223–31.

28. Ziboh VA, Miller CC, Cho Y. Metabolism of polyunsaturated fatty acids by skin epidermal enzymes: generation of antiinflammatory and antiproliferative metabolites. Am. J. Clin. Nutr. 2000 Jan;71(1 Suppl):361S–6S.

29. Owen RW, Giacosa A, Hull WE, Haubner R, Würtele G, Spiegelhalder B, et al. Olive-oil consumption and health: the possible role of antioxidants. Lancet Oncol. 2000 Oct;1:107–12.

30. Carrillo P. Propiedades del aceite de oliva en el mantenimiento de la integridad cutánea. Seminario médico. 2009;61(2):61–90.
