# Supplementary material for: Topical Olive Oil Is Not Inferior to Hyperoxygenated Fatty Aids to Prevent Pressure Ulcers in High-Risk Immobilised Patients in Home Care. Results of a Multicentre Randomised Triple-Blind Controlled Non-Inferiority Trial
Source: PLoS One. 2015 Apr 17;10(4):e0122238. doi: 10.1371/journal.pone.0122238 (PMC4401455; doi:10.1371/journal.pone.0122238)
Supplement: S1 CONSORT Checklist — (PDF) [file pone.0122238.s002.pdf]

Checklist of Items for Reporting Noninferiority or Equivalence Trials

Paper Section and	Item	Descriptor (Adapted for Noninferiority or Equivalence Trials)	Pages
**Title and abstract**	1*	How participants were allocated to interventions (eg, "random allocation," "randomized," or "randomly assigned"), *specifying that the trial is a noninferiority or* [illegible]	1-2
**INTRODUCTION**			
**Background**	2*	Scientific background and explanation of rationale, *including the rationale for using a noninferiority or equivalence design.*	3-5
**METHODS**			
Participants	3*	Eligibility criteria for participants *(detailing whether participants in the noninferiority or equivalence trial are similar to those in any trial[s] that established efficacy of the reference treatment) and the settings and locations where the data were collected.*	5-6
Interventions	4*	Precise details of the interventions intended for each group, detailing whether the reference treatment in the noninferiority or equivalence trial is identical (or very similar) to that in any trial(s) that established efficacy, and how and when they were actually administered.	7-8
Objectives	5*	Specific objectives and hypotheses, including the hypothesis concerning noninferiority or equivalence.	5-6
Outcomes	6*	Clearly defined primary and secondary outcome measures, detailing whether the outcomes in the noninferiority or equivalence trial are identical (or very similar) to those in any trial(s) that established efficacy of the reference treatment and, when applicable, any methods used to enhance the quality of measurements (eg, multiple observations, training of assessors).	7
Sample size	7*	How sample size was determined, detailing whether it was calculated using a noninferiority or equivalence criterion and specifying the margin of equivalence with the rationale for its choice. When applicable, explanation of any interim analyses and stopping rules (and whether related to a noninferiority or equivalence hypothesis).	7-8
**Randomization**			
Sequence generation	8	Method used to generate the random allocation sequence, including details of any	7
Allocation concealment	9	Method used to implement the random allocation sequence (eg, numbered	7
Implementation	10	Who generated the allocation sequence, who enrolled participants, and who	7
Blinding (masking)	11	Whether or not participants, those administering the interventions, and those assessing the outcomes were blinded to group assignment. When relevant, how the [illegible]	7
Statistical methods	12*	Statistical methods used to compare groups for primary outcome(s), specifying whether a 1- or 2-sided confidence interval approach was used. Methods for additional analyses, such as subgroup analyses and adjusted analyses.	8
**Results**			
Participant flow	13	Flow of participants through each stage (a diagram is strongly recommended). Specifically, for each group report the numbers of participants randomly assigned, receiving intended treatment, completing the trial protocol, and analyzed for the [illegible]	17
Recruitment	14	Dates defining the periods of recruitment and follow-up.	7
Baseline data	15	Baseline demographic and clinical characteristics of each group.	19
Numbers analyzed	16*	Number of participants (denominator) in each group included in each analysis and whether "intention-to-treat" and/or alternative analyses were conducted. State the results in absolute numbers when feasible (eg, 10/20, not 50%).	Fig 1
Outcomes and estimation	17*	For each primary and secondary outcome, a summary of results for each group and the estimated effect size and its precision (eg, 95% confidence interval). For the outcome(s) for which noninferiority or equivalence is hypothesized, a figure showing [illegible]	21
Ancillary analyses	18	Address multiplicity by reporting any other analyses performed, including subgroup analyses and adjusted analyses, indicating those prespecified and those exploratory.	
Adverse events	19	All important adverse events or side effects in each intervention group.	10
**Comment**			

Interpretation	20*	Interpretation of the results, taking into account the noninferiority or equivalence hypothesis and any other trial hypotheses, sources of potential bias or imprecision and the dangers associated with multiplicity of analyses and outcomes.	10-13
Generalizability	21	Generalizability (external validity) of the trial findings.	
Overall evidence	22	General interpretation of the results in the context of current evidence.	
