# Supplementary material for: Topical Olive Oil Is Not Inferior to Hyperoxygenated Fatty Aids to Prevent Pressure Ulcers in High-Risk Immobilised Patients in Home Care. Results of a Multicentre Randomised Triple-Blind Controlled Non-Inferiority Trial
Source: PLoS One. 2015 Apr 17;10(4):e0122238. doi: 10.1371/journal.pone.0122238 (PMC4401455; doi:10.1371/journal.pone.0122238)
Supplement: S1 Informed Consent — (PDF) [file pone.0122238.s003.pdf]

ANEXO 6.-

CONSENTIMIENTO INFORMADO.
Formulario de Información y Consentimiento Informado del Paciente.

ESTUDIO
Efectividad de la crema de aceite de oliva en la prevención de las úlceras por presión en pacientes inmovilizados en Atención Primaria.

Usted ha sido invitado a participar en un estudio de investigación. Antes de tomar una decisión es importante que usted entienda por qué el estudio se va a realizar y qué implicará. Por favor, lea la siguiente información de forma cuidadosa.

¿Quién está organizando y financiando la investigación y en qué consiste este estudio?

Este estudio ha sido financiado por el Ministerio de Sanidad y está siendo coordinado por Dª Inmaculada Lupiañez Pérez del Centro de Salud de Carlinda perteneciente a la Unidad de Investigación del Distrito Sanitario Málaga.
El Comité de Ética e Investigación Málaga Nordeste ha aprobado este proyecto.

El propósito de esta investigación es comprobar la efectividad de una nueva intervención que consiste en la aplicación de crema elaborada a base de aceite de oliva para prevenir la aparición de la úlcera por presión en pacientes inmovilizados.

Se aplicará crema elaborada a base de aceite de oliva en un grupo de pacientes y paralelamente se aplicará ácidos grasos hiperoxigenados en otro grupo con el que se comparan los resultados.

Este estudio será llevado a cabo en otros centros de salud de forma simultánea. Se pretende que participen 720 usuarios de Centros de Salud.

¿Por qué yo he sido elegido y porqué es importante que participe?

En Málaga se han seleccionado algunos Centros de Salud. Usted también ha sido seleccionado a participar voluntariamente por estar incluido entre los pacientes inmovilizados de esos centros.

Los resultados de esta investigación serán más válidos y fiables si todas las personas a las que invitamos a participar deciden tomar parte en el estudio y por ello esperamos y le pedimos que usted lo haga.

Depende de usted decidir si toma parte o no. Este estudio se realiza con la participación voluntaria de los entrevistados. Por lo tanto, usted es libre de retirarse en cualquier momento sin necesidad de dar ninguna razón.

¿En qué consiste mi participación en el estudio?

Usted será invitado a participar en el estudio a través de su médico de familia o enfermera de familia.

Si usted está de acuerdo en participar en el estudio, deberá firmar la hoja de consentimiento informado y entregarla a su enfermera.

¿Es este estudio confidencial?

Toda la información que usted facilita es totalmente confidencial. Los resultados del estudio se presentarán de manera que no revelarán la identidad de las personas participantes. Aunque se debe recoger el nombre y la dirección de cada persona para concertar las visitas, a cada persona que tome parte en el estudio, se le asignará un número que será el que figure en cada uno de los cuestionarios cumplimentados. Los registros que incluyen los nombres de las personas y las direcciones serán destruidos al finalizar el estudio. Por tanto, se garantiza que toda la información recogida será considerada totalmente confidencial. Únicamente si la enfermera cuando le hace la visita detectara que usted tiene un grave peligro para su salud, le pediría permiso para comunicárselo a su médico (1).

¿Cómo informarse sobre el estudio?

Si usted no está seguro sobre qué hacer, o desea más información, puede hablar con su Enfermera ó Médico de Familia del Centro de Salud. Si posteriormente, usted tiene más dudas, puede consultarlas con el investigador coordinador del estudio de campo, cuyos datos son Inmaculada Lupiañez Pérez.
(1). En cumplimiento de lo dispuesto en la Ley Orgánica 15/1999 de 13 de diciembre de Protección de Datos de Carácter Personal, la Unidad de Efectividad e Investigación del Distrito Sanitario Málaga, le informa que sus datos personales obtenidos o recogidos en su historia de salud, serán incorporados y protegidos para su tratamiento a un fichero automatizado.

Asimismo, se le informa que la recogida y tratamiento de dichos datos tiene como finalidad el estudio científico del que ha sido informado/a y para el cual ha prestado su consentimiento. De conformidad con la Ley 34/2002 y la Ley Orgánica 15/1999 puede ejercitar los derechos de acceso, rectificación, cancelación y oposición de sus datos dirigiendo un escrito a la Unidad de Efectividad e Investigación del Distrito Sanitario Málaga.

Consentimiento Informado del Paciente

ESTUDIO
Efectividad de la crema de aceite de oliva en la prevención de las úlceras por presión en pacientes inmovilizados en Atención Primaria. Ensayo clínico aleatorizado por conglomerados.

Por favor señale la casilla

1.- Confirmo que he entendido el propósito del estudio y he tenido la oportunidad de realizar preguntas.	☐
2.- Entiendo que mi participación en el estudio es voluntaria y que soy libre de abandonar el estudio en cualquier momento, sin dar ninguna razón y sin que mis derechos como paciente y de atención médica se vean afectados.	☐
3.- Estoy de acuerdo en que recojan información sobre mi historia de salud mientras participe en el estudio.	☐
4.- Yo estoy de acuerdo en tomar parte en este estudio.	☐

(EN MAYÚSCULAS)
Nombre del participante…………………...

D.N.I..

NUHSA:……………………………………….

Fecha.. Firma:……

Nombre de la enfermera……...

Fecha.. Firma.......
